# Supplementary material for: Inflammatory factors and risk of lung adenocarcinoma: a Mendelian randomization study mediated by blood metabolites
Source: Front Endocrinol (Lausanne). 2024 Aug 27;15:1446863. doi: 10.3389/fendo.2024.1446863 (PMC11384989; doi:10.3389/fendo.2024.1446863)
Supplement: Supplementary file 1 [file DataSheet1.zip › supplementary files/S2/GCST90200829/sensitivity-analysis.pdf]

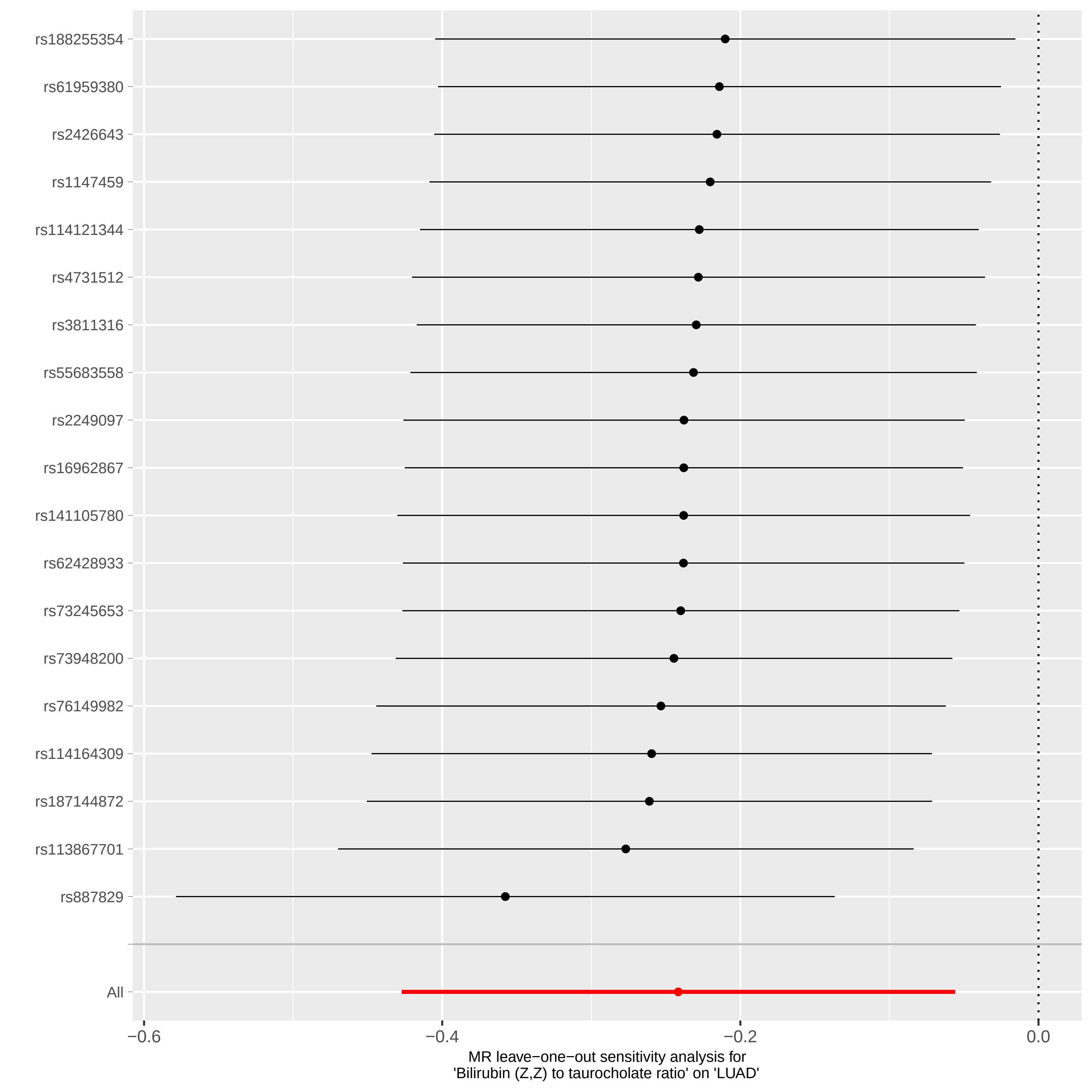

rs188255354
rs61959380
rs2426643
rs1147459
rs114121344
rs4731512
rs3811316
rs55683558
rs2249097
rs16962867
rs141105780
rs62428933
rs73245653
rs73948200
rs76149982
rs114164309
rs187144872
rs113867701
rs887829
All
−0.6
−0.4
−0.2
0.0
MR leave−one−out sensitivity analysis for
'Bilirubin (Z,Z) to taurocholate ratio' on 'LUAD'
